# Supplementary material for: A Critical View of the Application of the APEX Software (Aqueous Photochemistry of Environmentally-Occurring Xenobiotics) to Predict Photoreaction Kinetics in Surface Freshwaters
Source: Molecules. 2019 Dec 18;25(1):9. doi: 10.3390/molecules25010009 (PMC7017383; doi:10.3390/molecules25010009)
Supplement: Supplementary file 1 [file molecules-25-00009-s001.zip › 3.2.4_gcc-4.4.0.zip!/3.2.4_gcc-4.4.0/3.2.4_gcc-4.4.0/doc/octave/html/interpreter/String-Objects.html]

String Objects - Untitled


Next: Data Structure Objects,
Previous: Missing Data,
Up: Built-in Data Types


---

#### 3.1.3 String Objects

A character string in Octave consists of a sequence of characters
enclosed in either double-quote or single-quote marks. Internally,
Octave currently stores strings as matrices of characters. All the
indexing operations that work for matrix objects also work for strings.

See Strings, for more information.
